# On mechanism behind UV-A light enhanced antibacterial activity of gallic acid and propyl gallate against *Escherichia coli* O157:H7

**DOI:** 10.1038/s41598-017-08449-1

**Published:** 2017-08-16

**Authors:** Qingyang Wang, Erick Falcao de Oliveira, Solmaz Alborzi, Luis J. Bastarrachea, Rohan V. Tikekar

**Affiliations:** 1 Department of Nutrition and Food Science, University of Maryland, College Park, MD 20742 USA; 2 Department of Food Science and Technology, University of California, Davis, CA 95616 USA

## Abstract

Possible mechanisms behind the enhanced antimicrobial activity of gallic acid (GA) and its ester propyl gallate (PG) in the presence of UV-A light against *Escherichia coli* O157:H7 were investigated. GA by itself is a mild antimicrobial and has a pro-oxidant ability. We found that the presence of UV-A light increases the uptake of GA by the bacteria. Once GA is internalized, the interaction between GA and UV-A induces intracellular ROS formation, leading to oxidative damage. Concurrently, GA + UV-A also inhibits the activity of superoxide dismutase (SOD), magnifying the imbalance of redox status of *E. coli* O157:H7. In addition to ROS induced damage, UV-A light and GA also cause injury to the cell membrane of *E. coli* O157:H7. UV-A exposed PG caused oxidative damage to the cell and significantly higher damage to the cell membrane than GA + UV-A treatment, explaining its higher effectiveness than GA + UV-A treatment. The findings presented here may be useful in developing new antimicrobial sanitation technologies for food and pharmaceutical industries.

## Introduction

Gallic acid (GA, 3,4,5-trihydroxyl-benzoic acid) is a polyhydroxyphenolic compound widely distributed in various plants, fruit and vegetables^1^, and is a Generally Recognized As Safe compound (GRAS) to humans^2^. A variety of biological activities of GA have been demonstrated, including antioxidant^3^, anti-inflammatory^4^, and anti-cancer^5^ effects. Furthermore, GA has been found to have mild antimicrobial effect by itself against a wide variety of planktonic bacteria, biofilm, and fungi^6–9^. Although it has been well known that GA provides efficient protection against oxidative damage, it also has been reported to have pro-oxidant potential due to its autoxidation in certain conditions, resulting in the generation of reactive oxidative species (ROS) such as hydroxyl radicals, hydrogen peroxide, and superoxide anion^3^. This ROS generation resulted from the pro-oxidant potential of GA has been regarded as one of the contributors to the antimicrobial activity of GA^10, 11^, and has also been associated with the ability of GA to induce apoptosis of different cell lines^12–16^. Besides the effect of ROS, previous studies also attributed the antimicrobial activity of GA to the cell membrane disintegration and consequent leakage of intracellular constituents of bacteria^6, 8^. Propyl gallate (Gallate acid propyl ester, PG), a derivative of GA is widely used as a synthetic antioxidant in processed foods, cosmetics, and food packaging materials to prevent rancidity and spoilage^17^. Previously, alkyl gallate such as PG has been found to work as antibacterial and antifungal agents, but the mechanism was not assumed to be ROS related^18, 19^.

Studies have shown that some mild antimicrobials have synergistic antibacterial effects when they are combined with physical intervention such as heat or acid treatment, even at doses that are generally not inherently effective^20^. Our recent study established a novel synergistic antimicrobial method in which the non-thermal UV-A light treatment and GA generated enhanced antibacterial activity against *E. coli* O157:H7^21^. However, the mechanism of this synergistic effect has not been fully explored. Previously, this antibacterial effect was attributed to the photo-irradiation of GA by UV-A light and the subsequent generation of reactive oxidative species (ROS), by recognizing GA as a photosensitizer. High concentrations of ROS, including oxygen radicals and reactive non-radicals can cause cellular damage^22^. Nakamura *et al*. studied the antimicrobial action of photo-irradiated GA (4 mg L^−1^) by LED (400 nm). Although it was demonstrated that hydroxyl radicals and other ROS formed by photo-oxidation of GA, the concentration was too low to be effective alone, and there was no direct evidence that these ROS species were responsible for the inactivation^11, 23^. Thus, there was a need for a study focused on understanding the mechanism behind the synergistic interaction between UV-A light and GA.

The objective of this study was to investigate the antimicrobial mechanism of action of the GA + UV-A simultaneous treatment, by analyzing GA uptake, intracellular ROS generation, enzyme inhibition, and membrane injury of *E. coli* O157:H7. In addition, the effect of solution pH and the presence of ethylenediaminetetraacetic acid (EDTA) on the antimicrobial activity of the GA + UV-A system was investigated. Since pyrogallol (Py) and propyl gallate (PG), derivatives of GA, are structurally similar to GA, the antimicrobial activity of these two compounds was also evaluated.

## Results

### Antimicrobial activity of GA + UV-A against *E. coli O157:H7*: effect of GA concentration, solution pH, structure of derivatives, and the presence of EDTA

**Figure 1:**
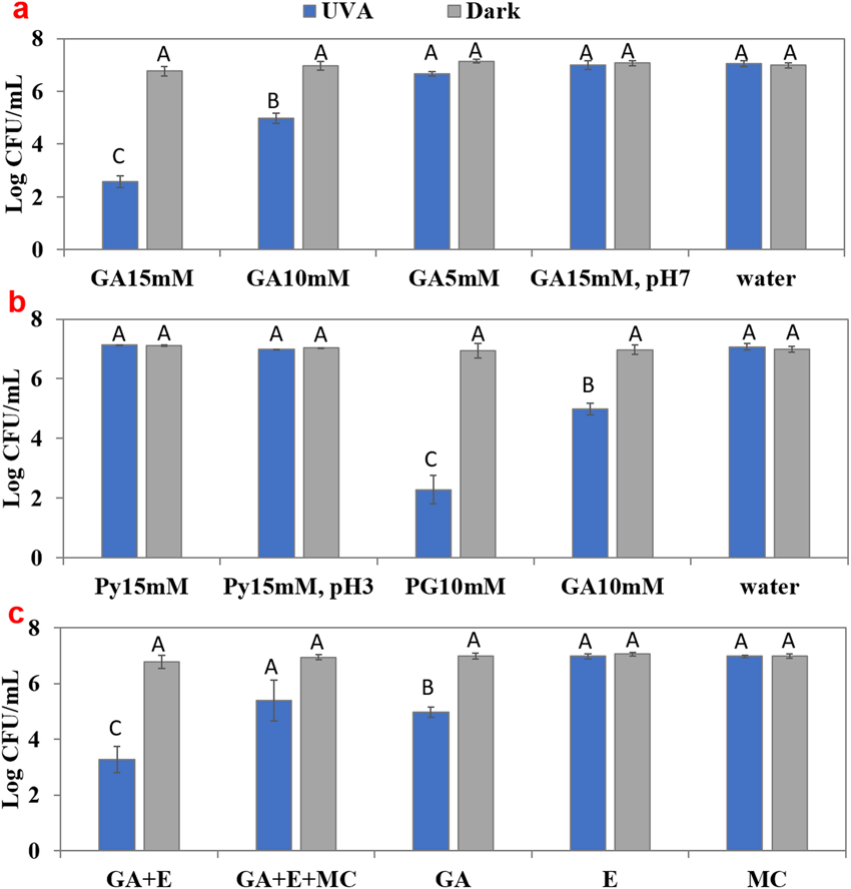
Evaluate the effect of GA concentration, solution pH, derivatives of GA and EDTA on the antimicrobial activity in the absence or presence of UV-A light. Logarithmic reduction of *E. coli* O157:H7 in the absence or presence of UV-A light and presence of (**a**) GA (0, 5, 10, 15 mM) and GA (15 mM) in phosphate buffer pH 7.4, (**b**) Pyrogallol (Py, 15 mM) or Propyl Gallate (PG, 10 mM), and (**c**) 15 mM GA + EDTA (E, 1 mM) with or without addition of 2 mM Mg^2+^ and 2 mM Ca^2+^ (MC). Mean ± SD. Means sharing the same letter are non-significant at *P* < 0.05 according to Tukey’s HSD test.

Figure 1a illustrates the antimicrobial activity of GA at various concentrations (5, 10 and 15 mM) dissolved in DI water or phosphate buffer (100 mM) at pH 7.4 in the absence or presence of UV-A light. GA alone in the absence of UV-A light and UV-A alone in the absence of GA did not show significant antibacterial effect (<0.5 log(CFU/mL)). In the presence of UV-A light, 5 mM GA caused a decrease of less than 1 log(CFU/mL) (0.41 ± 0.12), and was not significantly different (*P* > 0.05) from that of control. At 10 or 15 mM, the reduction in *E. coli* O157:H7 increased to 2.06 ± 0.19 and 4.41 ± 0.21 log(CFU/mL), respectively. The dependence of microbial inactivation on the concentration of GA was consistent with our previous study using synergistic interaction of GA and UV-A light against *E. coli* O157:H7 in simulated fresh produce^21^. Interestingly, GA + UV-A did not show significant (*P* > 0.05) antibacterial activity when the solution was prepared in phosphate buffer of pH 7.4.

To investigate if derivatives of GA had comparable antibacterial activity when treated under UV-A light, we performed similar experiments with Py and PG (Fig. 1b). Py + UV-A (15 mM) treatment did not cause any reduction of *E. coli* O157:H7 at either its natural pH (pH = 5.5) or at a pH similar to 15 mM GA (pH = 3.1). In contrast, PG + UV-A (10 mM) caused more than 6 log(CFU/mL) reduction, indicating a stronger antibacterial activity than GA + UV-A.

Figure 1c shows the effect of addition of 1 mM EDTA to GA (10 mM) solution. While 10 mM GA with UV-A light caused a 2.31 ± 0.57 log(CFU/mL) reduction, addition of 1 mM EDTA to 10 mM GA significantly (*P* < 0.05) increased the microbial inactivation to 3.64 ± 0.48 log(CFU/mL) (*P* < 0.05) in the presence of UV-A light. When a mixture of 2 mM CaCl_2_ and 2 mM MgCl_2_ was added to the bacteria solution containing 1 mM EDTA and 10 mM GA and exposed under UV-A light for 30 min, a microbial reduction of 1.56 ± 0.74 log(CFU/mL) was observed. This was significantly (*P* < 0.05) lower than that in the absence of metal ions, and not significantly (*P* > 0.05) different from the logarithmic reduction obtained from the 10 mM GA + UV-A treatment.

### Analysis of GA uptake by *E. coli O157:H7*

**Figure 2:**
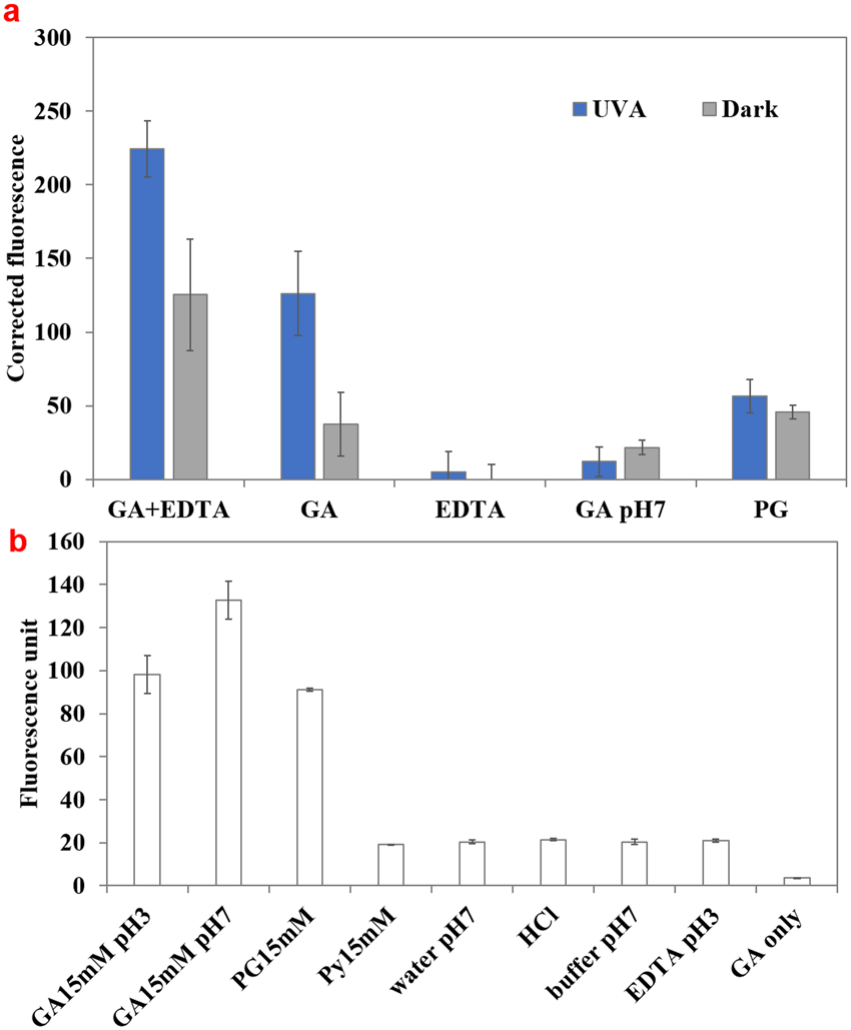
Measurement of uptake of gallic acid (GA) and its derivatives in *E. coli* O157:H7 as measured by binding with DPBA. (**a**) *E. coli* O157:H7 treated by GA (15 mM) + EDTA (1 mM) solution, GA (15 mM), EDTA (1 mM), GA in phosphate buffer solution (100 mM, pH 7.4), or PG (10 mM) in the presence and absence of UV-A light. Absolute fluorescence values were corrected by subtracting the fluorescence values for samples incubated in water and in dark. (**b**) The assessment of DPBA sensitivity to binding various compounds in the absence of bacteria. Mean ± SD.

Diphenylboric acid 2-aminoethyl ester (DPBA) is a flavonoid specific dye that becomes fluorescent upon conjugation with flavonoid compounds^24^. In the present study, it was used to detect the uptake of GA in *E. coli* O157:H7. Higher fluorescence intensity indicates higher association of GA with bacteria. Figure 2a shows that *E. coli* O157:H7 treated with 15 mM GA + UV-A light had significantly (*P* < 0.05) higher level of fluorescent intensity than that incubated in the dark, suggesting UV-A exposure increased the uptake of GA. Also, *E. coli* O157:H7 treated by GA + EDTA showed higher fluorescence intensity than by GA itself, suggesting GA uptake increased in the presence of EDTA. Fluorescence intensity within *E. coli* O157:H7 treated by GA in neutral pH (pH = 7.4) was not significantly different (*P* > 0.05) from that of control, indicating GA was not uptaken at pH 7.4. The GA uptake results were consistent with the inactivation results. It should be noted that although fluorescence intensity in *E. coli* O157:H7 treated by GA + EDTA incubated in the dark was not significantly different from that treated by GA + UV-A (*P* > 0.05), GA + EDTA without UV-A reduced the microbial population by 0.14 ± 0.24 log(CFU/mL), while GA + UV-A treatment had a 2.3 ± 0.57 log(CFU/mL) reduction. Thus, the extent of uptake may not be the only factor that affects the antibacterial effect of the treatment. It should also be noted that bacteria treated by PG + UV-A (10 mM) did not show a higher fluorescence intensity than that from GA + UV-A, although the antimicrobial activity of PG + UV-A was significantly (*P* < 0.05) higher than that of GA + UV-A exposure, indicating that inactivation of the bacteria by PG + UV-A followed a different mechanism than GA + UV-A. To verify the specificity of the complexation between DPBA and GA, experiments were also performed by measuring only the intensity of DPBA dissolved in water, HCl (pH = 3.1), phosphate buffer, EDTA (pH = 3.1), and GA in acidic or neutral pH without bacteria. The results (Fig. 2b) showed the relative affinity of DPBA to selected compounds or solvents. When GA of the same concentration was dissolved in a neutral pH buffer, the DBPA intensity was higher than that in acidic solution, indicating that GA was still able to complex with DBPA at neutral pH. Thus, the low fluorescence intensity in bacterial samples exposed to GA in phosphate buffer was due to the low extent of uptake of GA by bacteria at neutral pH. Therefore, the uptake of GA by bacteria was an important factor for GA to exert antibacterial activity with UV-A light. Similarly, PG was also able to bind with DPBA to produce fluorescent signal. However, based on the results shown in Fig. 2a, where fluorescence intensity from PG + UV-A treatment was significantly lower (*P* < 0.05) than that of GA + UV-A or GA + EDTA + UV-A, it is likely that the mechanism of inactivation for PG is distinct from GA. Py by itself did not show significant affinity to DPBA, therefore the uptake of Py by bacteria was not detected.

### Analysis of intracellular oxidative stress

**Figure 3:**
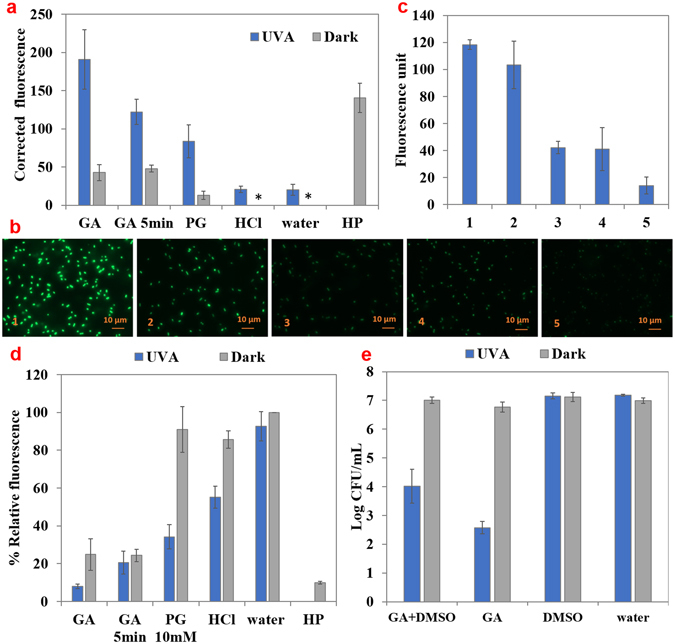
Measurement of oxidative stress experienced by bacteria by the select treatments. (**a**) Detection of reactive oxidative species (ROS) within *E. coli* O157:H7 using CellROX® Reagent Green upon treatment by GA (15 mM), PG (10 mM), HCl (pH 3.1), water, or hydrogen peroxide (HP, 1.5%), in the absence or presence of UV-A light for 30 or 5 min. Absolute fluorescence values were corrected by subtracting the fluorescence values for samples incubated in water and in dark. *Indicates that corrected fluorescence value was zero. (**b**) Fluorescent microscopy images (×100) of bacteria incubated with CellROX® Reagent Green and (1) 15 mM GA + UV-A, (2) 15 mM GA in dark, (3) HCl (pH = 3.1) + UV-A, (4) HCl (pH = 3.1) in dark, (5) DI water in dark. (**c**) Average maximum fluorescence intensity within the bacteria imaged using fluorescence microscopy. (**d**) Measurement of free thiols content from *E. coli* O157:H7 suspensions treated by GA (15 mM), PG (10 mM), HCl (pH 3.1), or water, with the presence or absence of UV-A light for 30 or 5 min. Hydrogen peroxide (HP, 1.5%), was used as a positive control. (**e**) Logarithmic reduction of *E. coli* O157:H7 by 15 mM GA + UV-A light, with or without 5% DMSO. Mean ± SD.

Generation of reactive oxygen species (ROS) in the bacteria is hypothesized to be one of the reasons for antimicrobial activity of the GA + UV-A treatment against *E. coli* O157:H7. To evaluate this hypothesis, we used a fluorescent probe, CellROX^®^ Green Reagent for measuring oxidative stress in cells^25–28^. Figure 3a shows the results from CellROX^®^ fluorescence intensity measurement. The higher fluorescence intensity indicates higher concentrations of intracellular ROS. Bacteria treated by hydrogen peroxide (1.5%) in dark for 30 min were used as the positive control. *E. coli* O157:H7 treated by 15 mM GA + UV-A light for 30 min had significantly (*P* < 0.05) higher intensity than UV-A alone or GA incubated in the dark, indicating higher concentration of ROS. Fluorescence intensity was also measured when the bacteria were exposed to a sub-lethal treatment of GA + UV-A for 5 min (<1 log(CFU/mL) reduction). A significant increase (*P* < 0.05) in fluorescence intensity was observed, for both GA in dark and GA + UV-A, indicating that the generation of ROS preceded the inactivation of the bacteria.

To visualize the ROS generated by GA + UV-A interaction, CellROX^®^ fluorescent reagent treated bacteria were observed under a fluorescence microscope (Fig. 3b) and the fluorescence intensity within bacteria was quantified (10 bacteria per treatment) in Fig. 3c. *E. coli* O157:H7 treated by GA + UV-A light had the highest fluorescent intensity, followed by bacteria exposed to GA in dark. HCl with or without UV-A light treated samples had similar and weaker fluorescent intensity than the treatment. Bacteria in DI water exhibited the weakest fluorescence. Thus, the fluorescence spectroscopic results and imaging results were qualitatively consistent.

Since the level of unoxidized free thiols is a good indicator for intracellular oxidative stress^29–32^, we measured the free thiols content in the cell (Fig. 3d) to further demonstrate that the oxidative stress was associated with bacterial inactivation. The relative percentage of free thiol in bacteria treated by GA + UV-A was 8.09 ± 1.24% (compared to control), which was significantly lower (*P* < 0.05) than that of the control (normalized to 100%). UV-A light, in the absence of GA only slightly and not significantly lowered (*P* > 0.05) the free thiol level (92.66 ± 7.78%). Bacteria incubated in HCl (pH = 3.1) in the dark had less free thiol content compared to control (85.66 ± 4.50%). However, when exposed to UV-A light for 30 min, HCl significantly lowered (*P* < 0.05) the thiol content to 55.13 ± 5.81%, but this content was still significantly higher (*P* < 0.05) than that of GA + UV-A treated sample. Thus, GA + UV-A caused more oxidative damage than that of the combination of low pH and UV-A. Results of the thiol content assay were consistent with results of CellROX^®^ assay. A sub-lethal treatment of GA + UV-A for 5 min lowered the free thiol content of *E. coli* O157:H7 to 20.64 ± 6.08% of control, indicating that thiol oxidation preceded inactivation. Thus, this result was also consistent with CellROX^®^ assay.

To further investigate if the ROS generated by GA + UV-A interaction is the main contributor to antimicrobial activity of this treatment, a known hydroxyl radical quencher, DMSO^33^ was added to GA solution to 5% v/v (Fig. 3e). In the presence of DMSO, GA + UV-A treatment caused a 3.12 ± 0.58 log(CFU/mL) reduction of *E. coli* O157:H7, approximately 1.5 log(CFU/mL) lower than that caused by GA + UV-A light without DMSO (4.41 ± 0.21 log(CFU/mL)). Although the presence of DMSO significantly (*P* < 0.05) attenuated the antibacterial efficacy, it did not fully eliminate it.

### Analysis of SOD activity

**Figure 4:**
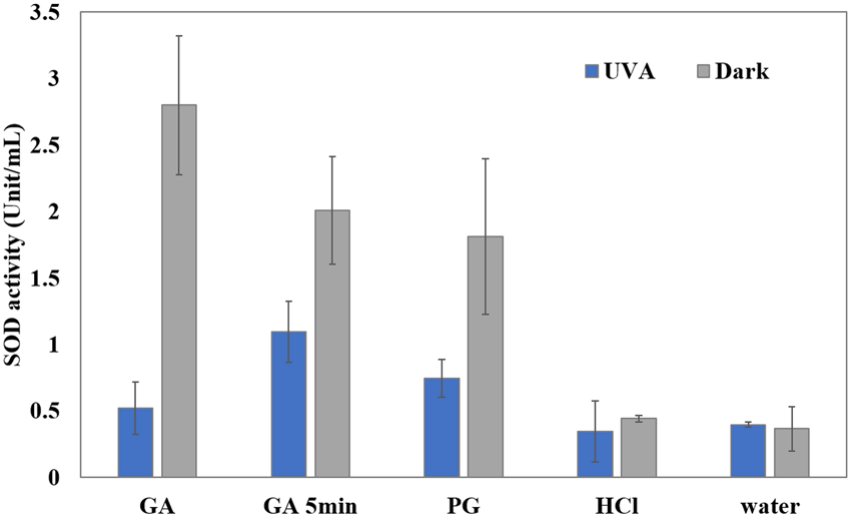
Superoxide dismutase activity (Unit/mL) of *E. coli* O157:H7 suspensions treated by UV-A light and selected compounds. *E. coli* O157:H7 was treated by GA (15 mM), PG (10 mM), HCl (pH = 3.1), or water for 30 or 5 min. Mean ± SD.

The activity of superoxide dismutase (SOD) within *E. coli* O157:H7 after various treatments is shown in Fig. 4. After incubation in GA (15 mM) for 30 min in the dark, the SOD activity within *E. coli* O157:H7 increased 7.6-fold (*P* < 0.05). However, when UV-A light was simultaneously present with GA, the SOD activity was low and the activity was not significantly different (*P* > 0.05) as control (bacteria treated in water in the dark for 30 min). Bacteria treated in HCl did not show significant increase in SOD activity either (*P* > 0.05). In a sub-lethal treatment of 5 min exposure, samples treated by GA alone had higher SOD activity than that subjected to UV-A light, which was consistent with the results of the 30-min treatment. Bacteria treated by PG (10 mM) in the dark for 30 min also showed increased SOD activity (4.95 fold than control) while treatment in the presence of UV-A showed diminished SOD activity, similar to GA + UV-A.

### Measurement of membrane damage

**Figure 5:**
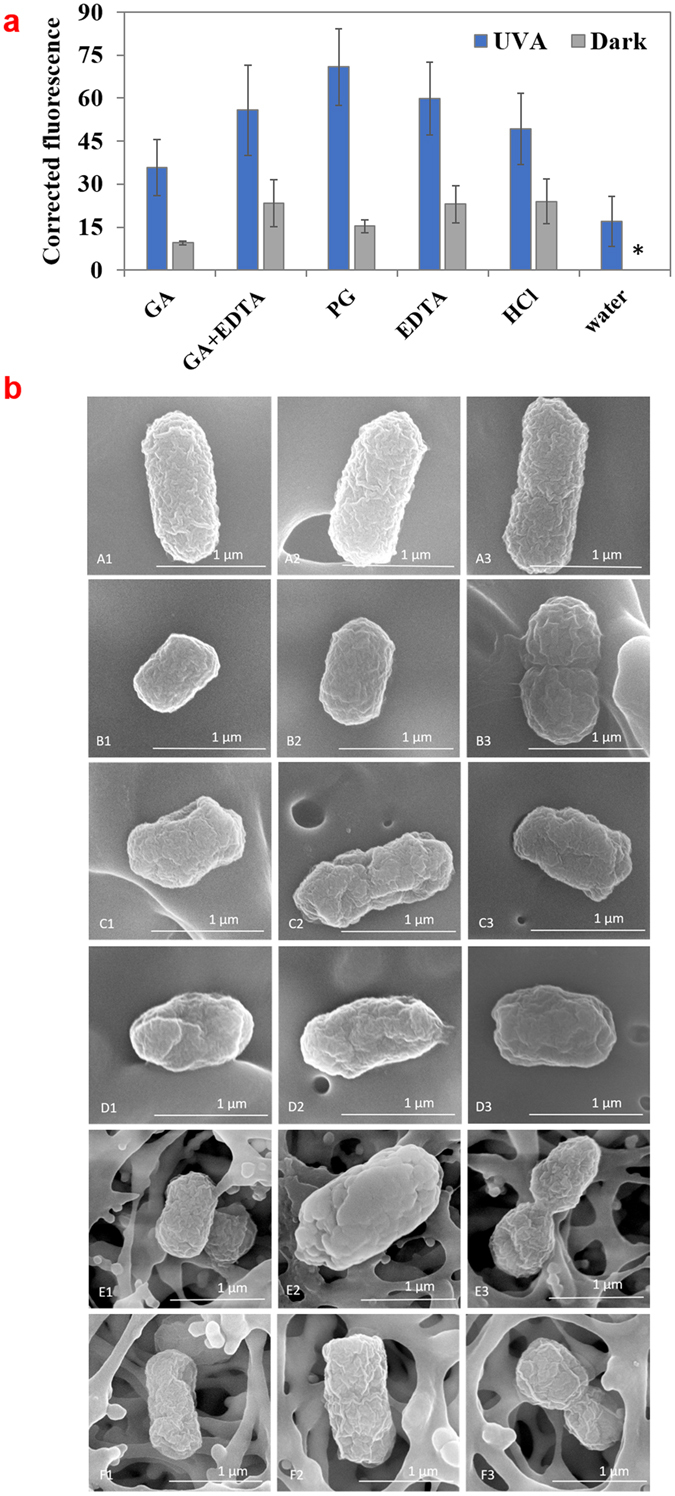
Analysis of cell membrane injury in *E. coli* O157:H7 treated by UV-A light with select compounds. (**a**) Permeability of *E. coli* O157:H7 to propidium iodide (PI) after treatment with GA (15 mM), GA (15 mM) + EDTA (1 mM), PG (10 mM), HCl (pH 3.1) or water for 30 min with or without UV-A exposure. Mean ± SD. Absolute fluorescence values were corrected by subtracting the fluorescence values for samples incubated in water and in dark. *Indicates that corrected fluorescence value was zero. (**b**) SEM images showing morphology of *E. coli* O157:H7 treated for 30 min by (A1-A3) water, (B1-B3) water + UV-A light, (C1-C3) 15 mM GA, (D1-D3) 15 mM GA + UV-A light, (E1-E3) 10 mM PG, (F1-F3) 10 mM PG + UV-A light.

*E. coli* O157:H7 membrane damage was assessed using fluorescent staining probe, propidium iodide (Fig. 5a). The presence of UV-A light significantly increased (*P* < 0.05) the fluorescence intensity for all the treatments, compared to the corresponding treatment without UV-A light exposure. The fluorescence intensity of bacteria treated by GA (15 mM) + UV-A light (35.74 ± 9.70) was significantly higher (*P* < 0.05) than that by GA incubated in the dark (9.50 ± 0.72) or control (corrected to 0), but not significantly different (*P* > 0.05) to UV-A alone (16.97 ± 8.64). The fluorescence intensity of GA increased in the presence of EDTA, and further increased significantly (*P* < 0.05) in the presence of UV-A light. PG (10 mM) + UV-A light treated sample had significantly higher (*P* < 0.05) fluorescence intensity than that of GA + UV-A. However, samples treated with EDTA or HCl also showed higher fluorescence intensity of PI in the presence UV-A, these treatments did not show significant (*P* > 0.05) antimicrobial effect. Therefore, membrane damage may only contribute partially to the bacterial inactivation.

Scanning electron microscopy (SEM) was used for surface morphology analysis of *E. coli* O157:H7 treated by GA + UV-A or PG + UV-A light (Fig. 5b). *E. coli* O157:H7 treated by 15 mM GA with or without UV-A light had shrinkage and irregular shape of the surface compared to control. However, the morphological difference between the bacteria treated with GA + UV-A or GA alone were visually comparable. *E. coli* O157:H7 treated by PG with or without UV-A light showed similar changes in morphology as those treated by GA + UV-A, indicating membrane damage occurred during this treatment as well.

## Discussion

The antimicrobial effect of GA + UV-A was dependent on the concentration of GA and the solution pH, and the effect was synergistic as neither UV-A light nor GA alone caused substantial inactivation. A previous study attributed the antimicrobial action of photoirradiated GA (4 mg/L) by LED (400 nm) to the generation of hydroxyl radicals and other ROS formed by photo-oxidation GA^11, 23^. They observed that approximately 7 µM hydrogen peroxide were generated and some lipid oxidation was also observed. However, the observed concentration of those ROS would be too low to be effective. Additionally, that study did not consider the location of ROS generation and fate of GA. Thus, more investigation was needed to understand the mechanism of inactivation.

An interesting observation was that although GA produced significantly more hydrogen peroxide upon exposure to UV-A light at pH 7.4 than at pH 3.1 (Supplementary Figure 1), the antimicrobial effect was negligible at pH 7.4 (Fig. 1a). A likely explanation is that at a neutral pH, the carboxyl group (pK_a_ = 4.0)^34^ was dissociated, conferring a net negative charge on the molecule and therefore decreasing its permeability within the bacterial membrane. Phenolic acids cross the cell membrane by passive diffusion in their undissociated form and the antibacterial activity of phenolic acid is dependent on the concentration of the undissociated acid^35, 36^. Thus, the uptake of the molecule by the bacteria is a pre-requisite for GA to exert its antimicrobial effect with UV-A light. Consistent with this postulation, GA in pH 7.4 solution did not show a significant uptake (Fig. 2a) and did not generate oxidative stress within the bacteria (Fig. 3). Py (15 mM) did not show any antibacterial activity at either natural (pH = 5.5) or acidic pH (pH = 3.1), probably due to its higher hydrophilicity that might reduce its affinity to cell membrane (Log P value of Py is 0.5^37^ while Log P value for GA is 0.7^38^), and thus reduce its uptake by bacteria. The importance of GA uptake within cell in bacterial inactivation was further highlighted by the effect of addition of EDTA in GA + UV-A system. EDTA permeabilizes outer membrane by binding divalent cations (in particular Mg^2+^ and Ca^2+^) that are essential for stabilizing the strong negative charges of the lipopolysaccharides (LPS) molecules^39–41^. Since cellular impermeability through the outer membrane is particularly important in the resistance of Gram-negative bacteria^41, 42^, EDTA enhanced the antibacterial efficacy of GA + UV-A treatment probably by disintegrating the outer membrane of bacteria and increasing the permeability of GA. This hypothesis was supported by the DPBA assay where GA uptake was shown to be significantly higher (*P* < 0.05) in the presence of EDTA, and the higher uptake of GA by EDTA under UV-A light exposure enhanced the antimicrobial effect (Figs 1c and 2a). Attenuation of EDTA effect by addition of excess Mg^2+^ and Ca^2+^ further validates this hypothesis.

CELLROX® is a cell permeable dye that can be oxidized by superoxide and hydroxyl radicals, inducing strong fluorescence in the presence of dsDNA^25^. CELLROX® assay and free thiols oxidation assay were both performed to corroborate the generation of ROS in *E. coli* O157:H7 as a result of GA + UV-A treatment (Fig. 3a and b). Results of these assays indicated that GA or PG induced higher ROS generation when UV-A was present than by themselves, even when the concentration of GA was reduced to a sub-lethal condition to the bacteria. The concentration of ROS detected in bacteria was consistent with the antimicrobial activity of the treatment. Since acidified water upon exposure to UV-A light did not significantly (*P* > 0.05) increase the ROS concentration, ROS produced within the bacteria were due to a specific interaction between GA and UV-A. The attenuation of antibacterial effect of GA + UV-A by DMSO, a hydroxyl radical scavenger (Fig. 3e) further supports the assumption that ROS were involved in the antimicrobial activity. Studies have shown that the generation of ROS may be the mechanism of action for some antibiotics^43^. Previous studies have also suggested that GA plays a role as a pro-oxidant inducing intracellular ROS generation and subsequent mammal cell apoptosis of a variety of cell lines and bacteria self-destruction^13, 44, 45^. Therefore, we inferred that the ROS observed in the present study may be produced directly by GA upon oxidation by UV-A light in bacteria (Supplementary Figure 1), or by indirectly mediating ROS formation through activating a variety of intracellular metabolic pathways. Also, GA as a weak organic acid dissociates when diffusing across membrane in the neutral pH environment of cytosol to protons and acid anion. The high anion accumulation may generate high turgor pressure and influence ROS generation, increasing oxidative stress^46^. When exposed to high levels of oxidative stress, bacteria can become more sensitive to other stresses such as low pH^47^, thus causing more damage to the cell.

In addition to intracellular ROS generation, the impairment of the redox detoxification and repair systems is another possible mechanism for bacteria to become more susceptible to oxidative attack^45^. Micromolar hydrogen peroxide is rarely lethal unless key oxidative defenses are also disabled^48^. It appeared that the simultaneous presence of GA and UV-A light inhibited the activity of SOD (Fig. 4), an essential enzyme for defending oxidative toxicity. GA is known to undergo autoxidation to produce ROS such as hydrogen peroxide and superoxide radicals^12, 49^. Therefore, it was reasonable to observe that SOD activity was significantly increased (*P* < 0.05) when GA was treated against *E. coli* O157:H7, indicating superoxide radicals were generated that activated SOD. This finding was consistent with the fact that SOD is strongly induced when *E. coli* is treated with antibiotics that generate intracellular superoxide^50^. However, when the bacteria were exposed to both GA and UV-A light, they showed lower activity of SOD than in the absence of UV-A light. The effect was evident in both sub-lethal and lethal treatment, but was far more pronounced at lethal treatment condition (30 min). It is likely that the amount of ROS produced by GA + UV-A treatment were too high to be quenched by SOD and related enzymes and these ROS also directly inactivated SOD, possibly through oxidation. In addition to this, a recent study demonstrated that activity of metabolic enzymes was also reduced by UV-A light and some organic acids including GA, indicating the reduction of metabolic activity and ATP level within bacterial cells could be other possible reasons for the antimicrobial effect of GA and UV-A treatment^51^.

Since GA may permeate outer membrane and trigger oxidative stress in bacteria, it is likely that cell membrane was damaged during the treatment. Previous studies have observed that GA and some other phenolic compounds disintegrate bacterial outer membrane^6, 8, 52, 53^. Results in Fig. 5a show that UV-A light alone caused membrane injury. The damage increased when UV-A was used in combination with GA, PG, EDTA, or HCl (pH = 3.1). However, it is interesting to observe that although the magnitude of membrane damage of *E. coli* O157:H7 was similar when treated by EDTA, PG, HCl, and EDTA + GA, only PG and GA + EDTA treatments caused substantial inactivation (>3 log(CFU/mL) in reduction) of *E. coli* O157:H7, suggesting the membrane damage by itself was not strongly correlated with the antimicrobial effect of the above treatments.

Although PG has a similar structure to that of GA except for the esterification of the carboxylic acid group, it exhibited characteristics distinct from that of GA in the absence and presence of UV-A light. PG showed higher antibacterial activity and lower uptake level than GA when UV-A was present. These differences can be attributed to its higher hydrophobicity (Log P for PG is 1.8^54^) due to the longer hydrocarbon tail on the carboxylic group that can increase its affinity to cell membrane. Thus, PG may preferentially localize within cell membrane than distribute within the cytoplasm. In the absence of UV-A light, PG had a marginal effect on ROS production (Fig. 3a), and had no effect on thiol oxidation (Fig. 3d), but increased SOD activity, similar to GA (Fig. 4). This finding is partially consistent with a previous study that showed the antimicrobial activity of alkyl gallates (in the absence of UV-A light) was not due to the ROS related pro-oxidant action, but likely came in part from the inhibition of the enzyme and membrane respiration chain by moving into the membrane lipid bilayer portions^18^. PG in combination with UV-A showed significantly elevated level of ROS compared to PG in dark (*P* < 0.05), had a similar impact on the SOD activity as GA + UV-A did, and a significantly higher (*P* < 0.05) membrane injury than GA + UV-A. Our results show that, in addition to ROS production and SOD inhibition, membrane injury was significantly enhanced (*P* < 0.05) by the simultaneous exposure to UV-A light and PG. Although antibacterial activity of PG is explored previously, albeit scarcely, its synergistic interaction with UV-A has not been reported before.

The biological damage caused by UV-A light is usually attributed to enhanced production of ROS that results in oxidative damage to lipids, proteins and DNA^55, 56^. Also, UV-A irradiation has been shown to cause membrane dysfunction and increase membrane permeability of bacteria^57^. In our experimental condition, UV-A light itself was not an effective bactericidal against *E. coli* O157:H7 (<1 log(CFU/mL) reduction). One role UV-A light played in the combination treatment of GA + UV-A was increasing the permeability of GA into the cells. Nevertheless, both EDTA and UV-A light increased the uptake of GA to the cell (Fig. 2a). However, EDTA and GA in the dark incubation did not have lethal effect. Only in the presence of UV-A light (GA + UV-A or GA + EDTA + UV-A treatment) could the internalized GA exert antibacterial activity. Therefore, the contribution of UV-A was more than increasing the GA uptake. It may have also increased the oxidative stress generated from GA, possibly through its photo-oxidation or altering the bacterial metabolism as discussed earlier.

Correlating the results from complimentary experiments, we propose that the antimicrobial mechanism of this combined treatment against *E. coli* O157:H7 is as follows: GA by itself is a mild antimicrobial and has a pro-oxidant ability. The presence of UV-A light increases the uptake of GA. Once GA is internalized, the interaction between GA and UV-A directly or indirectly induces intracellular ROS formation, leading to oxidative damage. Concurrently, the activity of ROS defending enzyme, such as SOD, is also inhibited, magnifying the oxidative damage to *E. coli* O157:H7. Other than oxidative stress, the acidification effect of GA and membrane damage of UV-A is also associated with the inactivation of *E. coli* O157:H7. It is also plausible that these combinations of stresses may have an impact on the bacterial DNA and metabolism. These complimentary stresses affect various aspects of cell metabolism and structure, ultimately leading to the death of bacteria. PG showed a stronger antimicrobial activity in the presence of UV-A light than GA + UV-A. In addition to the generation of oxidative stress, a higher level of bacterial membrane damage was responsible for the antimicrobial effect of PG + UV-A treatment.

## Methods

### Bacteria cultivation

A Shiga toxin negative *E. coli* O157:H7 (ATCC #700728, Manassas, VA) was kindly provided by Prof. N. Nitin at University of California-Davis. The bacteria were cultured in Tryptic Soy Broth (TSB) at 37 °C for 20 h to obtain the bacterial population in stationary phase before each experiment.

### GA + UV-A treatment

The overnight bacterial culture was diluted in sterilized GA solution of various concentrations (5, 10, 15 mM) prepared in either DI water or phosphate buffer (100 mM, pH = 7.4), with or without the presence of EDTA (1 mM) to reach a final concentration of approximately 1 × 10^7^ CFU/mL. To evaluate the antimicrobial activity of some GA derivatives, the culture of bacteria was also exposed to pyrogallol (Py, 15 mM) or propyl gallate (PG, 10 mM). Following that, 2 mL of each bacterial suspension was transferred to a well of a 6-well flat bottom polystyrene plate, and exposed to UV-A light for 30 min. The UV-A light source (Spectroline^TM^, Westbury NYUSA) was a bench-top, batch type chamber with a peak wavelength of 365 nm and average intensity of 3425 µW/cm^2^ applied at the surface from a distance of 17 cm. Bacterial suspensions incubated with select compounds and stored in the dark for 30 min were used as controls. After the treatment, the bacteria suspensions were serially diluted in 0.2% (w/v) buffered peptone water, and an aliquot of 100 µL suspensions from each dilution was transferred and plated onto Tryptic Soy Agar (TSA, Difco^TM^, Detroit MI USA) plates. The plates were incubated at 37 °C for 24 h before enumeration.

### Analysis of GA uptake by *E. coli* O157:H7

The uptake of GA was evaluated using diphenylboric acid 2-aminoethyl ester (DPBA), a flavonoid specific dye that becomes fluorescent upon conjugation with flavonoid compounds and can permeate the bacterial membrane. However, we found that it also showed increased fluorescence upon binding with GA and its derivatives. A volume of 1 mL of overnight culture of *E. coli* O157:H7 was transferred to a 1.5 mL Eppendorf tube and centrifuged twice at 10,000 rcf for 2 min to obtain a pellet (~ 1 × 10^9^ CFU/mL). A volume of 1 mL of solution of GA (15 mM) + EDTA (1 mM), GA (15 mM), EDTA (1 mM), GA in phosphate buffer solution (100 mM, pH = 7.4), or PG (10 mM) was added to the pellet and mixed. To evaluate the effect of pH on GA permeability, GA (15 mM) in phosphate buffer at the pH of 7.4 was also used to treat the pellet. DI water was added to the pellet as control. Then, 2 mL of the suspension was transferred to a 6-well plate and exposed to UV-A light for 30 min as described previously. Control samples were treated in the exact same manner except for the UV-A treatment. After the incubation, each suspension was transferred back to an Eppendorf tube and centrifuged at 10,000 rcf for 2 min. The supernatant was removed and the pellet was washed twice with DI water. Then, 450 µL of DPBA solution (0.2% w/v in DMSO) was added to the pellet and mixed. The final suspension of 100 µL was transferred to a 96-well plate and fluorescence intensity was measured using a SpectraMax M5e plate reader (Molecular Devices LLC, Sunnyvale CA) at excitation/emission wavelength of 405/465 nm. The fluorescence intensity ratio was corrected using the following equation:

1 $$\begin{array}{c}Corrected\,fluorescence\\ \quad =fluorescence\,intensit{y}_{treatedsample}-\,fluorescence\,intensit{y}_{control}\end{array}$$

### Analysis of intracellular oxidative stress

The intracellular oxidative stress of *E. coli* O157:H7 was analyzed using two distinct approaches. CellROX^®^ Green Reagent is a novel fluorogenic probe for oxidative stress measurement in live cell. The cell-permeant dye is weakly fluorescent while in a reduced state and exhibits bright green photostable fluorescence upon oxidation by reactive oxidative species (ROS) and subsequent binding to DNA. A volume of 1 mL of overnight culture of *E. coli* O157:H7 at stationary phase was transferred to an Eppendorf tube, washed with DI water, centrifuged for 2 min at 10,000 × g, and exposed to UV-A or kept under the dark with GA (15 mM), PG (10 mM), GA in phosphate buffer (pH = 7.4), HCl (pH = 3.1), or DI water. Hydrogen peroxide (HP, 1.5%), was used as a positive control. After the treatment, CellROX^®^ Green probe was added to the bacterial suspension at a concentration of 5 µM and incubated at 37 °C for 30 min without light exposure. Then, the bacterial suspension was washed three times with sterile phosphate buffered saline (PBS) solution, and re-suspended in 500 µL of PBS solution. This final suspension was transferred either to a 96-well plate for intensity measurements at 485/520 (ex/em), or to a fluorescence microscope for imaging. The fluorescence intensity was corrected using equation (1).

For fluorescence imaging, 5 µL of the stained bacteria suspension was placed between a slide and a cover slip for microscopy evaluation. Zeiss Axio Observer Z1 fluorescence microscope (Oberkochen, Germany) at University of Maryland (Department of Microbiology) was used for observation of the stained bacteria at a magnification of 100×. The light source was a TL Halogen lamp. Filter wavelength of excitation/emission were 450–495/500–550 respectively. The fluorescence intensity of images were measured using image processing software ImageJ^58^ after the images were set to identical threshold.

The intracellular oxidative stress was also analyzed by measuring free thiols in bacteria using Thiol Detection Assay Kit (Cayman Chemical Company, Ann Arbor, MI). A volume of 1 mL of overnight culture was transferred to a sterile Eppendorf tube, washed with DI water, centrifuged for 2 min at 10,000 × g, and treated with or without the exposure of UV-A light in the presence of GA (15 mM), PG (10 mM), HCl (pH = 3.1). Bacterial pellet suspended in DI water only without UV-A light treatment was set as the control. Hydrogen peroxide (1.5%), was used as a positive control. After the treatment and a wash with DI water, 1 mL of cold lysis buffer (Tris-HCl 10 mM with EDTA 1 mM) was added to the pellet and mixed thoroughly. Then, 500 µL of suspension was transferred to a new Eppendorf tube containing approximately 400 µL of silica beads. The mixture was vortexed for 10 min before centrifuging at 15,000 rcf at 4 °C. The supernatant was diluted 5-fold in thiol assay buffer included by the assay kit. Finally, 50 µL of diluted supernatant were transferred to a 96-well plate, to which 50 µL of thiol fluorometric detector were added. The plate was incubated under the dark for 5 min, before measuring fluorescence at the excitation and emission wavelength of 385 and 515 nm respectively. The fluorescence intensity was normalized using the following equation:

2 $$ \% \,Relative\,fluorescence=\,\frac{\,fluorescence\,intensit{y}_{treatedsample}}{\,fluorescence\,intensit{y}_{control}}\times 100$$

### Analysis of superoxide dismutase activity

Activity of Superoxide dismutase (SOD) within *E. coli* O157:H7 was analyzed using Superoxide dismutase assay kit according to the manufacturer’s protocol (Cayman Chemical, MI). *E. coli* O157:H7 suspensions were diluted in 15 mM GA, 10 mM PG, HCl with the same pH of 15 mM GA, or DI water as control. After incubation in the presence or absence of UV-A light, the samples were washed with DI water. Then, samples were homogenized in 20 mM HEPES buffer (pH = 7.2, containing 1 mM EGTA, 210 mM mannitol, and 70 mM sucrose), followed by centrifugation at 1,500 × g for 5 min at 4 °C, and recovery of the supernatant. To quantify SOD activity, 200 µL of diluted SOD radical detector (included in the assay kit), 10 µL of sample, and 20 µL of diluted xanthine oxidase (included in the assay kit) were successively added to a 96-well plate, covered with the plate’s lid, and incubated on a shaker for 30 min to mix at room temperature. The absorption was analyzed using a SpectraMax M5e plate reader (Molecular Devices LLC, Sunnyvale CA) at a wavelength of 440 nm. The SOD activity was determined by referring to a SOD activity standard curve established using the same assay kit.

### Analysis of membrane damage

Membrane damage of *E. coli* O157:H7 during the treatment was analyzed by using the fluorescence probe propidium iodide (PI) and through scanning electron microscope (SEM). PI is a red-fluorescent nuclear and chromosome counterstain that penetrates only bacteria with damaged membranes and is frequently used to detect cell membrane damage^59, 60^. Test solutions consisting of bacteria (~1 × 10^9^ CFU/mL) suspended in GA solution (15 mM), PG solution (10 mM), EDTA solution (1 mM), or HCl solution (pH = 3.1) were treated under UV-A light exposure or incubated under the dark for 30 min. Bacteria in DI water alone in the dark was used as live control. After treatment, samples were washed with DI water and centrifuged for 2 min at 10,000 × g. Then, a volume of 50 µL of PI was added to each sample to reach a concentration of 5 µM, following dark incubation at room temperature for 15 min. Subsequently, samples were washed and suspended in 500 µL 1 × PBS. A volume of 100 µL of this sample were transferred to a 96-well plate, and the fluorescence intensity was measured using a plate reader with excitation and emission wavelength of 490/635 nm. The fluorescence intensity was corrected using equation (1).

Membrane damage was also visualized by SEM imaging based on method described by Kihm *et al*.^61^. *E. coli* O157:H7 (approximately 1 × 10^7^ CFU/mL) suspended in 15 mM GA solution, 10 mM PG solution or DI water were exposed to UV-A light or incubated in the dark for 30 min. Then, cells were recovered by filtering through a 0.2 µm sterile filter, fixed by incubating in 0.25% glutaraldehyde for 1 h, rinsed three times in DI water, dehydrated six times in ethanol of increasing concentration, and stored in a desiccator overnight for dehydration prior to imaging. To observe cell morphology under SEM, bacteria were coated with gold (20 nm) with a sputter coater. After coating with gold, the morphology of bacteria was studied using a SEM at an accelerating voltage of 10 kV.

### Statistical analysis

Experiments were performed in triplicate. Statistical analysis of the data was performed using the two-tailed unpaired *t*-test (*α* = 0.05) by Microsoft Excel 2016 (Microsoft Inc., Redmond WA, USA). When appropriate, statistical significance was determined through analysis of variance performed by JMP 13.1.0 (SAS Institute Inc., Cary NC, USA) followed by pairwise comparisons through Tukey’s HSD test (*α* = 0.05).

## Electronic supplementary material

Supplementary information
