# Supplementary material for: On mechanism behind UV-A light enhanced antibacterial activity of gallic acid and propyl gallate against Escherichia coli O157:H7
Source: Sci Rep. 2017 Aug 16;7:8325. doi: 10.1038/s41598-017-08449-1 (PMC5559599; doi:10.1038/s41598-017-08449-1)
Supplement: Supplementary file 1 — Supplementary information [file 41598_2017_8449_MOESM1_ESM.pdf]

Supplementary information

On mechanism behind UV-A light enhanced antibacterial activity of gallic acid and propyl gallate against *Escherichia coli* O157:H7

Qingyang Wang[1], Erick Falcao de Oliveira [2], Solmaz Alborzi[1], Luis J. Bastarrachea[1], Rohan V. Tikekar*[1]

[1] Department of Nutrition and Food Science, University of Maryland, College Park, MD 20742, USA

[2] Department of Food Science and Technology, University of California, Davis, CA 95616, USA

Methods

Evaluating the generation of hydrogen peroxide from (gallic acid) GA exposed to UV-A light

The generation of hydrogen peroxide was investigated by ferrous ion oxidation xylenol orange (FOX) method with some modifications [1]. It is based on the ability of hydrogen peroxide to convert ferrous ions into ferric ions which can form a complex with xylenol orange (XO), the concentration of which is determined using spectrophotometry. GA solutions were prepared by dissolving 0.5 mM GA in distilled water (pH 3.3) or phosphate buffer (100 mM, pH 7.4). DI water without GA was used as control. An aliquot (5 mL) of each test solution was transferred to a crystallizing dish (KIMAX®, NJ, USA) and exposed to UV-A light or incubated in dark for 15 min. Samples were obtained periodically for hydrogen peroxide measurement. FOX assay reagent of 50 µL containing 1 mM xylenol orange, 2.5 mM ferrous sulfate, 1 M sorbitol, and 250 mM sulfuric acid was added to 350 µL samples, followed by incubation at room temperature for 30 min. After that, the absorbance of solutions was measured at 560 nm using a Spectroscopy M5e plate reader (Molecular Devices LLC, Sunnyvale CA). Hydrogen peroxide concentrations were determined based on an external standard curve (**Supplementary figure 2**).

Results and Discussion

Supplementary figure 1 shows the generation of hydrogen peroxide from GA solution in natural or modified neutral pH, in the presence or absence of UV-A light. In 5 min, GA in pH 7.4 solution in presence UV-A light generated significantly ($P<0.05$) higher amount of hydrogen peroxide than other samples did, followed by GA in neutral pH incubated in dark. GA in pH 3.3 with UV-A light exposure also generated more hydrogen peroxide than GA without UV-A. Water by itself did not generate hydrogen peroxide with or without UV-A. This result suggests neutral pH environment enhance autoxidation process of GA and more hydrogen peroxide is generated. This is consistent with previous studies that autoxidation of GA proceeds and behaves differently with higher pH values [2,3]. Our result also suggests that UV-A light increased the amount of hydrogen peroxide generated by GA, probably due to oxidation.

Reference

1. Jiang, Z. Y., Woollard, a C. & Wolff, S. P. Hydrogen peroxide production during

experimental protein glycation. *FEBS Lett.* **268,** 69–71 (1990).

2. Nikolić, G., Veselinović, A., Mitić, Ž. & Živanović, S. HPLC - DAD Study of Gallic Acid Autoxidation in Alkaline Aqueous Solutions and the Influence of Mg (II) Ion. *Acta Fac. Medicae Naissensis* **28,** 219–224 (2011).

3. Eslami, A. C., Pasanphan, W., Wagner, B. a & Buettner, G. R. Free radicals produced by the oxidation of gallic acid: An electron paramagnetic resonance study. *Chem. Cent. J.* **4,** 15 (2010).

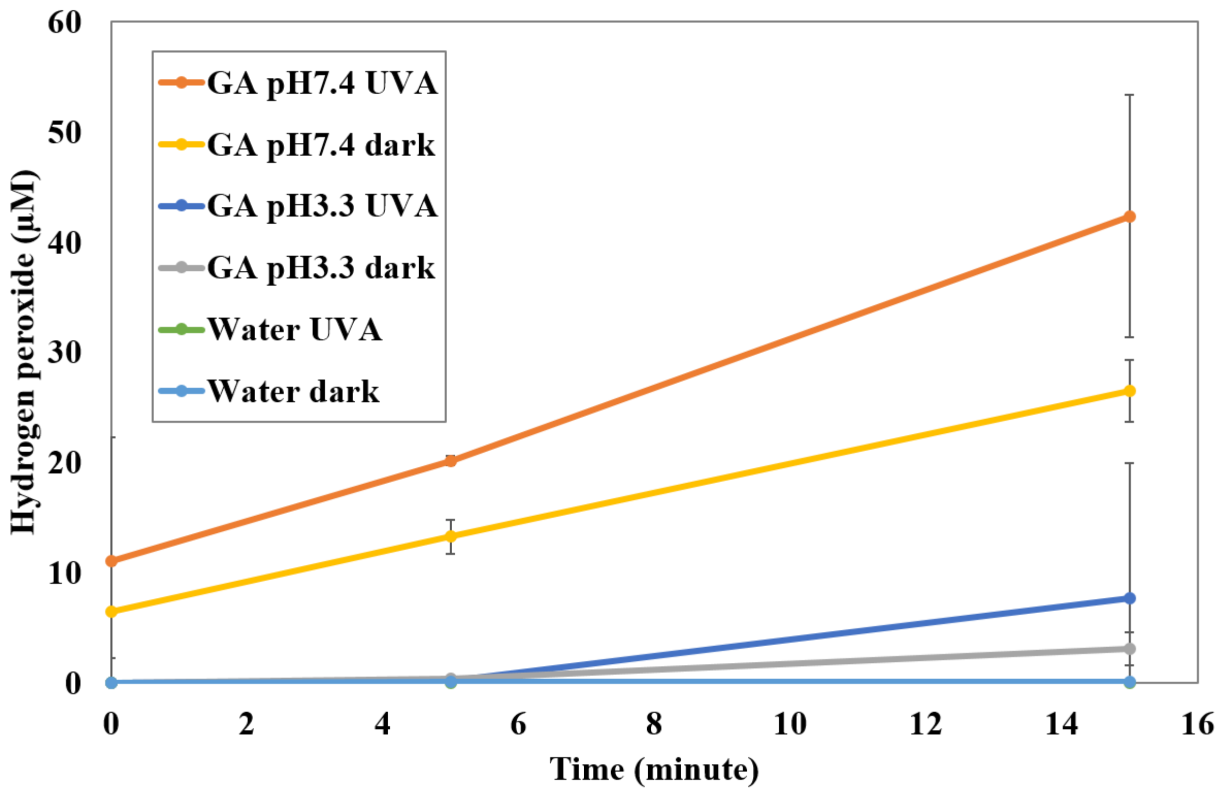

Supplementary figure 1. Generation of hydrogen peroxide from GA (0.5 mM) in DI water or phosphate buffer (100 mM, pH 7.4), in the presence or absence of UV-A light exposure. Mean ± SD.

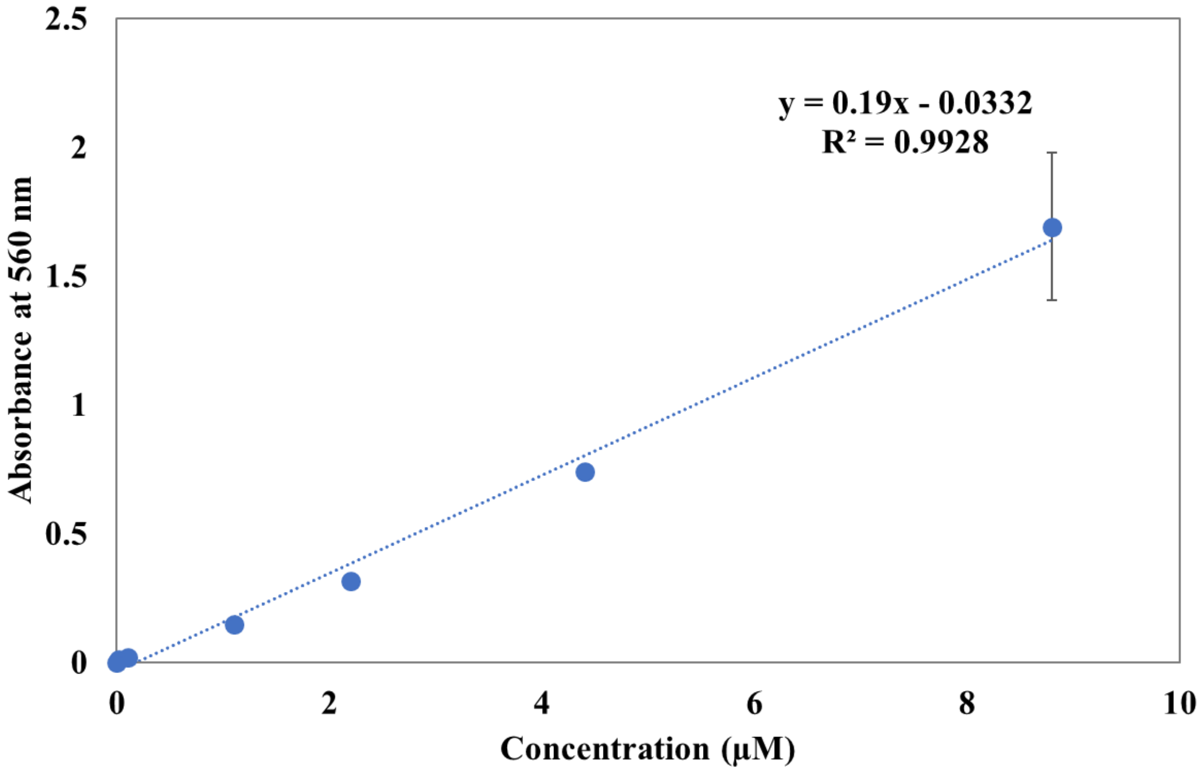

Supplementary figure 2. FOX assay standard curve for hydrogen peroxide measurement. Hydrogen peroxide was serial diluted at room temperature (24 °C) and the absorbance was measured by UV-Vis spectrophotometer.
